# Associations of Violence Against Women With Comorbid Symptoms of Depression and Anxiety Among Left-Behind Women in Rural China: Cross-Sectional Study

**DOI:** 10.2196/72064

**Published:** 2025-08-07

**Authors:** Leying Hou, Zeyu Luo, Weidi Sun, Jiayao Ying, Jing Wu, Shiyi Shan, Wen Liu, Peige Song

**Affiliations:** 1 Center for Clinical Big Data and Statistics of the Second Affiliated Hospital Zhejiang University School of Medicine, School of Public Health Zhejiang University School of Medicine, No. 866 Yuhangtang Road, Xihu District, Hangzhou City, Zhejiang Province, Hangzhou, 310058, China, 86 88981368; 2 Department of General Practice and Primary Care, The University of Melbourne, Melbourne, Victoria, Australia

**Keywords:** left-behind women, violence, depressive and anxiety symptoms, resilience, social support

## Abstract

**Background:**

Violence against women (VAW) is a major public health and human rights concern with profound mental health consequences. However, the association between specific VAW forms and mental health, particularly among left-behind women in rural China, remains unclear.

**Objective:**

This study aimed to identify the associations of VAW with depression, anxiety, and comorbid symptoms and to explore the potential roles of resilience and social support.

**Methods:**

The cross-sectional survey was conducted in Y City, Henan Province, China, in July 2023. A multistage stratified random sampling method was used to recruit left-behind women, resulting in a final sample of 1503 participants. Data on participants and their VAW were collected through a face-to-face questionnaire survey. The forms of VAW assessed were nonpartner violence (NPV) and intimate partner violence (IPV; including remote IPV). Depressive symptoms were evaluated using the 10-item Center for Epidemiological Studies Depression Scale, while anxiety symptoms were assessed with the Generalized Anxiety Disorder-7. The comorbid symptoms of depression and anxiety (CDA) were ascertained as the simultaneous presence of depressive and anxiety symptoms. A multivariable logistic regression model was used to estimate the odds ratio and 95% CIs. A 4-way decomposition analysis was conducted to test the mediation roles and interactions of resilience and social support between VAW and mental health outcomes. Population attributable fractions and pathway-specific population attributable fractions were calculated to estimate the burden of mental health outcomes attributable to VAW.

**Results:**

Lifetime VAW (adjusted odds ratio [aOR] 1.84, 95% CI 1.32‐2.54) was associated with an increased risk of CDA. Women who were exposed to lifetime IPV (aOR 1.84, 95% CI 1.32‐2.56), remote IPV (aOR 2.79, 95% CI 1.60‐4.74), and NPV (aOR 2.63, 95% CI 1.58‐4.26) had an increased likelihood of reporting CDA. Similar associations could also be found for depressive symptoms and anxiety symptoms. In the 4-way decomposition analysis for VAW and CDA, mediation effects of low resilience and social support were statistically significant (*P*<.05), whereas none of the interactions reached significance (*P*>.05). The pure mediation proportion was 28.2% for the low resilience and 18.6% for the social support between VAW and CDA. A total of 20.8% of CDA cases, 15.1% of depressive symptoms cases, and 22.7% of anxiety symptoms cases were attributable to VAW. Among these, low resilience accounted for 7.2% and low social support accounted for 4.7% of CDA cases as mediators.

**Conclusions:**

Lifetime VAW, including IPV (and remote IPV) and NPV, shows significant associations with CDA and depressive and anxiety symptoms among rural left-behind women in China. The associations are partly mediated by low resilience and social support. Targeted strategies, including efforts to reduce violence against rural left-behind women, enhance their resilience and strengthen their social support networks, are urgently needed.

## Introduction

With rapid urbanization and economic reforms, rural left-behind women—those whose husbands migrate to urban areas for employment—are becoming increasingly common in many low- and middle-income countries [1-3]. In China, over 50 million women are left behind in rural areas due to internal migration [4]. This demographic shift is shaped by structural inequalities, such as women’s disadvantaged position, underdeveloped social services, and cultural norms like familism, which often prioritize family needs over individual desires [5]. As a result, left-behind women bear a “triple burden” of caring for children, supporting older family members, and managing agricultural responsibilities in the absence of their spouses [6]. The prolonged separation from their spouses, combined with socioeconomic pressures, raises concerns about their mental health [3].

Mental disorders, particularly depression and anxiety, have become increasingly prevalent and are now major global mental health concerns [7]. Previous studies showed that depression and anxiety frequently co-occur, sharing common symptoms and risk factors like adverse life events [8]. Comorbid symptoms of depression and anxiety (CDA) are more debilitating than either condition alone, leading to greater psychosocial impairment and poorer health outcomes [9]. Among left-behind women, these disorders are of particular concern due to the compounded stressors they face. The absence of a spouse, social isolation, and the potential for increased economic pressure can create an environment that fosters psychological distress [10,11]. Thus, attention to the mental health of left-behind women is essential, particularly given the limited availability of mental health services in rural settings [12].

A crucial, yet often overlooked, determinant of mental health is violence against women (VAW). VAW is a pervasive global issue encompassing physical, emotional, and sexual abuse, with intimate partner violence (IPV) being the most commonly studied form [13,14]. Although the physical absence of a husband might suggest reduced exposure to IPV, left-behind women are susceptible to experiencing remote IPV, a form of abuse facilitated by technology, where geographically distant partners exert control through harassment, threats, or emotional abuse via phone calls, messaging, or social media [15,16]. Moreover, nonpartner violence (NPV) should be given attention among left-behind women since they experience prolonged periods of living alone or with other family members [17,18]. A growing body of literature demonstrates that exposure to VAW, particularly IPV, is strongly associated with adverse mental health outcomes, including depression and anxiety [19,20]. For example, for women in Afghanistan under the Taliban government, exposure to traumatic events such as violence can contribute to depressive and anxiety symptoms, ultimately diminishing their quality of life [21-23]. However, significant gaps remain in our understanding of how different forms of VAW, specifically IPV, remote IPV, and NPV, impact mental health outcomes among left-behind women.

Resilience is the ability to adapt positively in the face of adversity [24]. Social support, similarly, is considered a positive role in improving mental health outcomes [25]. Despite their known protective effects, the roles of resilience and social support as mediators or moderators in the relationship between VAW and mental health, particularly among rural left-behind women, remain unclear.

**Figure 1.**
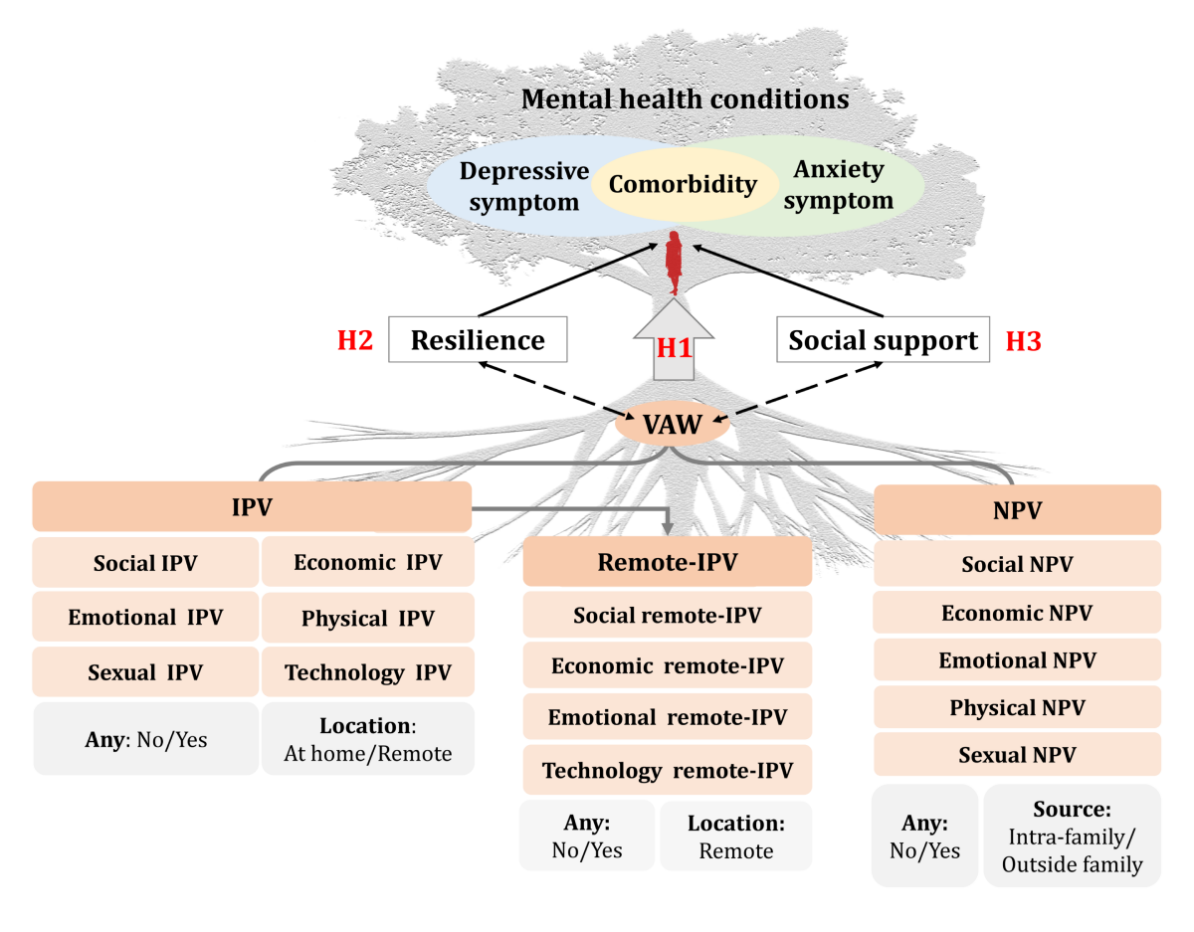
Conceptual framework illustrating the hypothesized associations between various forms and types of violence and mental health outcomes among rural left-behind women in China, with resilience and social support as potential mediators. In this figure, the tree trunk represents mental health status composed of depressive and anxiety symptoms, while the roots symbolize various forms of violence as underlying factors. The tree as a whole illustrates how these root-level exposures may influence the development of mental health outcomes. H1: hypothesis 1; H2: hypothesis 2; H3: hypothesis 3; IPV: intimate partner violence; NPV: nonpartner violence; VAW: violence against women.

This study aimed to address these gaps by investigating the associations of VAW with depressive, anxiety, and comorbid symptoms among left-behind women in rural China, and to explore the potential roles of resilience and social support. Specifically, this study sought to test the following three primary hypotheses (Figure 1):

1. Hypothesis 1 (H1): VAW is positively associated with depressive and anxiety symptoms, with different forms, types, and periods of violence exhibiting various associations.
2. Hypothesis 2 (H2): Resilience serves as a mediator and moderates the association between VAW and mental health conditions.
3. Hypothesis 3 (H3): Social support acts as a mediator and moderates the association between VAW and mental health conditions.

## Methods

### Study Design and Participants

The cross-sectional survey was conducted in Y City, Henan Province, China, in July 2023, where there is frequent labor migration to urban areas for work (Figure S1 in Multimedia Appendix 1). For participant recruitment, a multistage stratified random sampling method was used. First, 7 towns were chosen: 3 low-, 2 medium-, and 2 high-economic towns. Second, 102 survey sites were selected within these towns. With support from local Women’s Federation staff or social workers of these selected survey sites, participants were invited to complete a face-to-face questionnaire administered by trained field investigators in meeting rooms of village committee service centers. Data on women’s sociodemographic and lifestyle information, violence exposure, and mental health outcomes were self-reported and collected following the World Health Organization recommendations to maximize participant safety and disclosure [26]. To ensure data quality, daily debriefing sessions were held with all field investigators to address potential questions arising from interviews based on their submitted questionnaires. The statistics team performed daily checks on antifraud questions, minimum completion time thresholds, and logical consistency checks (eg, implausible age values).

Eligible participants were rural married women aged 18 years or older with normal communication and understanding ability, whose husbands had been absent for at least 6 months within the past year. A total of 1525 questionnaires were finally retained for the survey, of which 1519 were valid. Participants with missing or ambiguous data on violence (n=3) or mental health outcomes (n=13) were excluded, resulting in a final sample of 1503 women (Figure S1 in Multimedia Appendix 1).

### Ethical Considerations

The study protocol was approved by the Ethics Review Committee of Zhejiang University School of Public Health (approval number: ZGL202306-5). All participants provided written informed consent for participation and publication. Study data were anonymized prior to analysis. Each participant received a health gift package valued at approximately ¥20 (approximately US $2.80). No identifiable images of participants are included in the manuscript or Multimedia Appendix files.

### Assessment of VAW

The questionnaire used to assess various forms of VAW in this study was developed based on the World Health Organization’s VAW Instrument [27]. The adapted questionnaire was validated through a 2-round Delphi survey with 18 experts, ensuring its credibility. The lifetime VAW was divided into 2 primary forms according to the sources: IPV (violence from partners) and NPV (violence from other family members or outside the family) (Figure 1). Given that technology-driven, geographically distant abuse is unique for rural left-behind women, remote IPV was specifically measured. IPV was measured using 25 items across 6 types (social IPV, economic IPV, emotional IPV, physical IPV, sexual IPV, and technology-facilitated IPV). Remote IPV was assessed using 16 items across 4 types (social remote IPV, economic remote IPV, emotional remote IPV, technology-facilitated remote IPV). NPV was measured through 19 items across 5 types (social NPV, economic NPV, emotional NPV, physical NPV, and sexual NPV) (Table S1 in Multimedia Appendix 1).

### Assessment of Mental Health Outcomes

Depressive symptoms were measured using the 10-item Center for Epidemiological Studies Depression scale [28], which demonstrated good internal consistency (Cronbach α=0.80) [29]. Participants were asked how frequently they experienced a series of depressive symptoms over the past week, with response options ranging from 0 (rarely or none of the time) to 3 (most or all of the time). The total 10-item Center for Epidemiological Studies Depression Scale score ranged from 0 to 30, with a cutoff score of 10 indicating the presence of depressive symptoms [30].

Anxiety symptoms were assessed using the generalized anxiety disorder-7 scale [31,32], which also showed excellent internal consistency (Cronbach α=0.92) [33]. The Generalized Anxiety Disorder-7 includes 7 items rated on a 4-point scale: 0 (not at all) to 3 (nearly every day), with a cutoff score of 5 indicating anxiety symptoms [34].

CDA was defined as the simultaneous presence of both depressive and anxiety symptoms. In this study, CDA, depressive symptoms, and anxiety symptoms were the primary mental health outcomes of interest.

### Assessment of Resilience and Social Support

Resilience was measured using the 10-item Connor-Davidson Resilience Scale [24,35,36], with a Cronbach α of 0.98. Items were rated on a 0‐4 scale, with higher scores indicating greater resilience (range 0‐40). Social support was measured using the Social Support Rating Scale, including objective support (3 items), subjective support (4 items), and utilization of support (3 items), with a total score of 66 points. The Social Support Rating Scale has demonstrated acceptable internal consistency and reliability, with a Cronbach α of 0.70 in this study [37].

### Ascertainment of Covariates

Covariates include women’s sociodemographic, lifestyle, and left-behind related variables. The age of women was assessed as a continuous variable. Education was classified into primary school and below, middle school, and high school and above. Work was categorized into no job, farmer, formal job, casual job, or others. Annual household income in CNY was divided into below ¥30,000, ¥30,001‐50,000, and ¥50,001 and above (all currency values in Chinese yuan [¥] are based on the conversion rate of ¥7.1 = US $1, the approximate exchange rate at the time of the study). An unhealthy lifestyle consists of smoking, drinking, and poor sleep quality. BMI was calculated as weight divided by the square of height and categorized into 3 levels (normal weight or under: <23.9 kg/m^2^, overweight: 23.9‐27.8 kg/m^2^, and obesity: ≥27.9 kg/m^2^) [38]. Chronic disease history was self-reported physician diagnosis of the common noncommunicable diseases, including hypertension, dyslipidemia, diabetes or hyperglycemia, gastrointestinal disorders, liver diseases, heart diseases, stroke, urological disorders, respiratory system diseases, arthritis, or rheumatism. Length of being left behind was divided into below 3 years, 3‐6 years, 6 years and above.

### Statistical Analysis

Descriptive statistics summarized rural left-behind women’s characteristics by CDA, depressive, and anxiety symptoms. Skewed continuous variables were reported as medians (IQRs) and compared via the Wilcoxon rank-sum test. Categorical variables were presented as frequencies (%) and analyzed using Pearson chi-square or Fisher exact tests.

Multivariable logistic regressions were used to analyze associations of any VAW and its forms (IPV [including remote IPV] and NPV) as well as specific types with CDA, depressive, and anxiety symptoms. Models were adjusted for age, education, occupation, household income, healthy lifestyle, BMI, chronic disease history, and length of being left behind. Restricted cubic splines were used to investigate the dose-response associations between the number of VAW items and mental health outcomes.

A 4-way decomposition analysis was performed to quantify how much of the association between VAW and mental health outcomes could be attributed to mediation (the sum of the mediated interaction and the pure indirect effect) or interaction (the sum of the reference interaction and the mediated interaction) of low resilience and social support [39]. In addition, separate mediation and additive and multiplicative interaction analyses were also performed. Detailed methods are described in Multimedia Appendix 1.

Population attributable fractions (PAFs) were calculated to estimate the proportion of CDA, depressive symptoms, and anxiety symptoms cases that could potentially be attributable to VAW based on the following equation[40]: $PAF=\frac{P_{e}(RR-1)}{1+P_{e}\left(RR-1\right)}$. Our study replaced risk ratios (RRs) in the formula with odds ratios (ORs) obtained in the above logistic regression. The fraction of participants being exposed to the risk factor (Pe) and the OR of the outcome associated with the corresponding factors were derived from the study. The 95% CI of PAF was calculated using bootstrap [41]. Furthermore, we explored pathway-specific PAFs of potential mediators between VAW and outcomes, which indicated the relative reduction in outcome prevalence if a certain mediating pathway was disabled. The “graphPAF” R package was used to obtain pathway-specific PAFs [42,43].

All statistical analyses were performed using R software (version 4.2.1; R Foundation for Statistical Computing). Statistical significance was considered to be <.05 with a 2-sided, or 95% CIs of OR that did not cross 1.00.

## Results

### Characteristics

The characteristics of rural left-behind women stratified by CDA are shown in Table 1. Of the 1503 rural left-behind women, 12.8% (n=192) had CDA. Women with CDA were more likely to experience VAW (43.8% vs 29.4%), IPV (40.1% vs 26.6%), remote IPV (12.0% vs 4.4%), and NPV (14.6% vs 5.9%), compared with those without CDA (all *P*<.05). For VAW types, similar comparisons are also evident.

**Table 1.** Characteristics of Chinese rural left-behind women by comorbid symptoms of depression and anxiety, based on a cross-sectional study conducted in China in 2023.

Characteristic	Overall (n=1503)	No (n=1311)	CDA^a^ (n=192)	*P* value^b^
Sociodemographic				
Age (year), median (IQR)	52.0 (43.0-57.0)	52.0 (43.0-57.0)	52.0 (42.0-56.3)	.97
Education, n (%)				.24
Primary school and below	526 (35.0)	449 (34.2)	77 (40.1)	
Middle school	788 (52.4)	693 (52.9)	95 (49.5)	
High school and above	189 (12.6)	169 (12.9)	20 (10.4)	
Occupation, n (%)				.20
No job	686 (45.6)	591 (45.1)	95 (49.5)	
Farmer or formal job	510 (33.9)	443 (33.8)	67 (34.9)	
Casual job or others	307 (20.4)	277 (21.1)	30 (15.6)	
Household income (¥)^c^, n (%)				.001
Below 30,000	296 (19.7)	244 (18.6)	52 (27.1)	
30,001‐50,000	531 (35.3)	456 (34.8)	75 (39.1)	
50,001 and above	676 (45.0)	611 (46.6)	65 (33.9)	
Smoking status, n (%)				.10
No smoking	1494 (99.4)	1305 (99.5)	189 (98.4)	
Smokers	9 (0.6)	6 (0.5)	3 (1.6)	
Drinking status, n (%)				.13
No drinking	1412 (93.9)	1227 (93.6)	185 (96.4)	
Drinkers	91 (6.1)	84 (6.4)	7 (3.6)	
Sleep quality, n (%)				<.001
Good	681 (45.3)	637 (48.6)	44 (22.9)	
Average	425 (28.3)	376 (28.7)	49 (25.5)	
Poor	397 (26.4)	298 (22.7)	99 (51.6)	
BMI (kg/m^2^), n (%)				.42
Normal weight and under (<23.9)	715 (47.6)	618 (47.1)	97 (50.5)	
Overweight (23.9‐27.8)	571 (38.0)	498 (38.0)	73 (38.0)	
Obesity (≥27.9)	217 (14.4)	195 (14.9)	22 (11.5)	
Chronic disease history, n (%)				<.001
No	902 (60.0)	812 (61.9)	90 (46.9)	
Yes	601 (40.0)	499 (38.1)	102 (53.1)	
Length of being left behind, n (%)				<.001
Below 3 years	244 (16.2)	195 (14.9)	49 (25.5)	
3‐6 years	241 (16.0)	206 (15.7)	35 (18.2)	
6 years and above	1,018 (67.7)	910 (69.4)	108 (56.3)	
Resilience scores, median (IQR)	41.0 (37.0-45.0)	41.0 (37.0-45.0)	38.0 (33.0-42.0)	<.001
Social support scores, median (IQR)	30.0 (23.0-36.0)	30.0 (25.0-36.0)	20.0 (16.0-26.0)	<.001
VAW^d^				
Any VAW, n (%)				<.001
No	1,033 (68.7)	925 (70.6)	108 (56.3)	
VAW	470 (31.3)	386 (29.4)	84 (43.8)	
VAW number, median (IQR)	0.0 (0.0-1.0)	0.0 (0.0-1.0)	0.0 (0.0-3.0)	<.001
Class of VAW number, n (%)				<.001
0	1,033 (68.7)	925 (70.6)	108 (56.3)	
1	196 (13.0)	177 (13.5)	19 (9.9)	
2	100 (6.7)	84 (6.4)	16 (8.3)	
≥3	174 (11.6)	125 (9.5)	49 (25.5)	
Any IPV^e^, n (%)				<.001
No	1,077 (71.7)	962 (73.4)	115 (59.9)	
IPV	426 (28.3)	349 (26.6)	77 (40.1)	
IPV type, n (%)				
Social IPV	88 (5.9)	63 (4.8)	25 (13.0)	<.001
Economic IPV	40 (2.7)	32 (2.4)	8 (4.2)	.17
Emotional IPV	189 (12.6)	148 (11.3)	41 (21.4)	<.001
Physical IPV	301 (20.0)	238 (18.2)	63 (32.8)	<.001
Sexual IPV	32 (2.1)	22 (1.7)	10 (5.2)	.01
Technology IPV	7 (0.5)	4 (0.3)	3 (1.6)	.05
Any remote IPV, n (%)				<.001
No	1422 (94.6)	1253 (95.6)	169 (88.0)	
Remote IPV	81 (5.4)	58 (4.4)	23 (12.0)	
Remote IPV type, n (%)				
Social remote IPV	50 (3.3)	34 (2.6)	16 (8.3)	<.001
Economic remote IPV	23 (1.5)	19 (1.4)	4 (2.1)	.52
Emotional remote IPV	26 (1.7)	20 (1.5)	6 (3.1)	.13
Technology remote IPV	7 (0.5)	4 (0.3)	3 (1.6)	.05
Any NPV^f^, n (%)				<.001
No	1398 (93.0)	1234 (94.1)	164 (85.4)	
NPV	105 (7.0)	77 (5.9)	28 (14.6)	
NPV type, n (%)				
Economic NPV	8 (0.5)	5 (0.4)	3 (1.6)	.07
Emotional NPV	89 (5.9)	62 (4.7)	27 (14.1)	<.001
Physical NPV	24 (1.6)	18 (1.4)	6 (3.1)	.11
Sexual NPV	1 (0.1)	1 (0.1)	0 (0.0)	>.99
Social NPV	2 (0.1)	2 (0.2)	0 (0.0)	>.99

a CDA: comorbid symptoms of depression and anxiety.

b *P* values represent statistical measurements of comparing “No” and “CDA” using Wilcoxon Rank Sum test, Pearson chi-square test, or Fisher exact test.

c Chinese ¥1=US $0.14.

d VAW: violence against women.

e IPV: intimate partner violence.

f NPV: nonpartner violence.

### Associations Between VAW and Mental Health Outcomes

**Figure 2.**
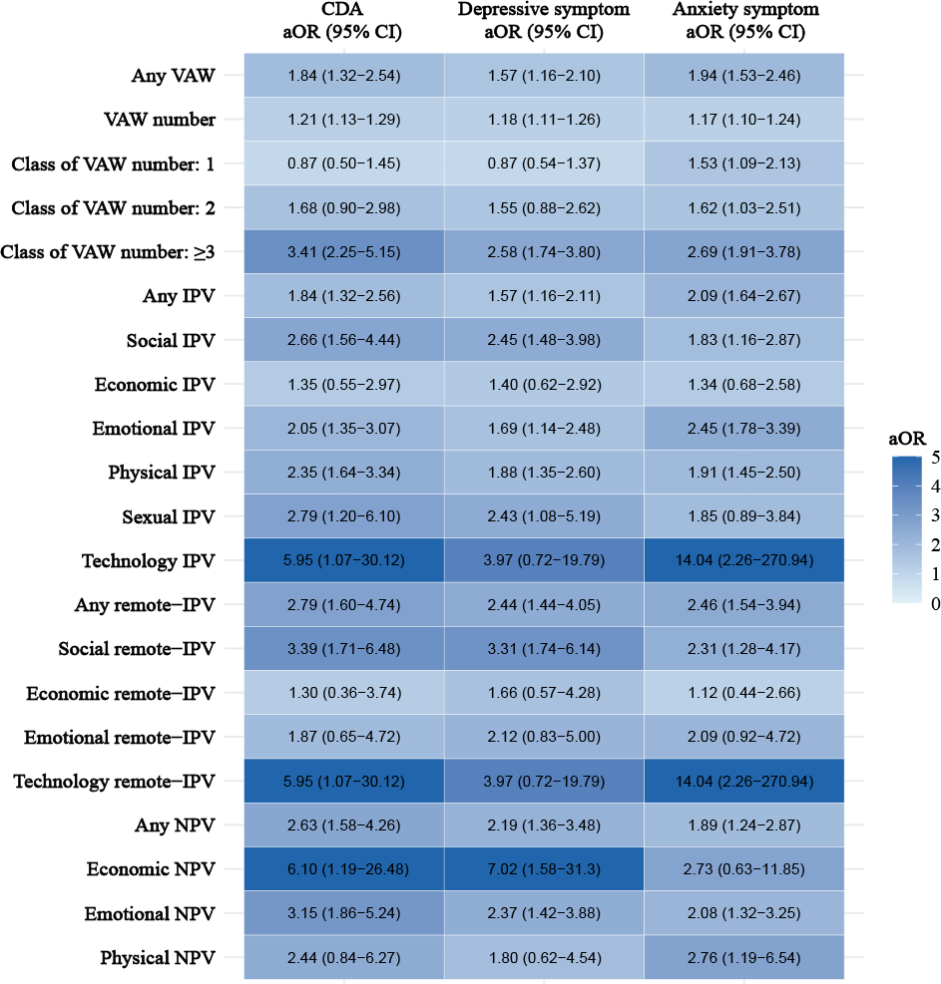
Heat maps of aOR from multivariable logistic regression analyses examining associations between various forms and types of violence and mental health outcomes among Chinese rural left-behind women, based on a cross-sectional study conducted in China in 2023. A darker color in a gradient from white to blue shows a higher aOR of corresponding outcomes. All models were adjusted for age, education, occupation, household income, unhealthy lifestyle (smoking, drinking, poor sleep quality), BMI, chronic disease history, and length of being left behind. aOR: adjusted odds ratio; CDA: comorbid symptoms of depression and anxiety; IPV: intimate partner violence; NPV: nonpartner violence; VAW: violence against women.

In the multivariable logistic models presented in Figure 2 and Table S2 in Multimedia Appendix 1, compared with no VAW, VAW exposure was associated with an increased CDA risk (adjusted odds ratio [aOR] 1.84, 95% CI 1.32‐2.54), and this risk rose with each additional VAW item (aOR 1.21, 95% CI 1.13‐1.29). When VAW was categorized by item count, women with 3 or more VAW items showed a markedly higher risk of CDA (aOR 3.41, 95% CI 2.25‐5.15). Similar associations were also observed for depressive and anxiety symptoms in relation to VAW (Figure 2 and Table S2 in Multimedia Appendix 1). Restricted cubic spline curves exhibited dose-response associations between increased VAW numbers and elevated risks of mental health outcomes (Figure S2 in Multimedia Appendix 1).

For the 3 forms of VAW, lifetime IPV (aOR 1.84, 95% CI 1.32‐2.56), remote IPV (aOR 2.79, 95% CI 1.60‐4.74), and NPV (aOR 2.63, 95% CI 1.58‐4.26) were all associated with higher odds of CDA. Among the types of IPV, only economic IPV did not show a significant association with mental health outcomes. Social remote IPV (aOR 3.39, 95% CI 1.71‐6.48), technology-facilitated remote IPV (aOR 5.95, 95% CI 1.07‐30.12), economic NPV (aOR 6.10, 95% CI 1.19‐26.48), and emotional NPV (aOR 3.15, 95% CI 1.86‐5.24) were all associated with higher odds of CDA. Similar findings were observed for depressive symptoms and anxiety symptoms, respectively (Figure 2 and Table S2 in Multimedia Appendix 1).

### Mediation and Interaction Effects

**Figure 3.**
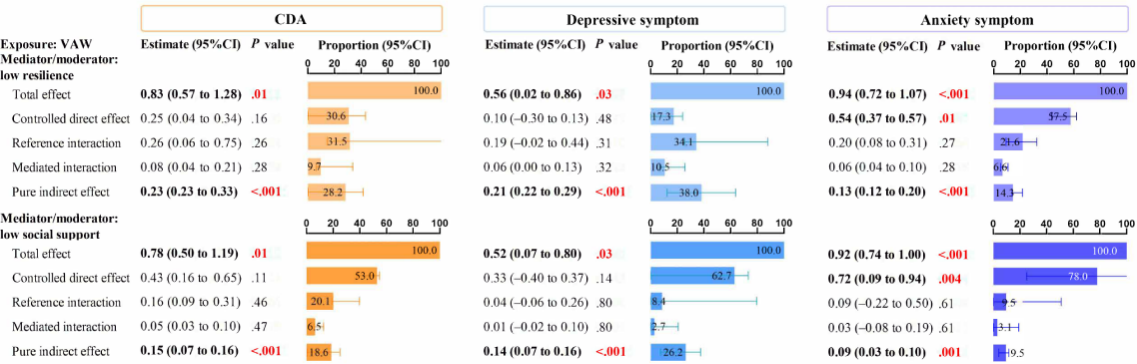
Four-way decomposition analysis of the associations between violence and mental health outcomes with low resilience or social support as mediators among Chinese rural left-behind women, based on a cross-sectional study conducted in China in 2023. Controlled direct effect indicates association not caused by mediation or interaction, reference interaction indicates association due to interaction only, mediated interaction indicates association due to mediation and interaction, and pure indirect effect indicates association due to mediation only. The total mediation proportion comprises the combined effects of the mediated interaction and the pure indirect effect. Similarly, the total interaction proportion is determined by the sum of the reference interaction and the mediated interaction. All models were adjusted for age, education, occupation, household income, unhealthy lifestyle (smoking, drinking, and poor sleep quality), BMI, chronic disease history, and length of being left behind. CDA: comorbid symptoms of depression and anxiety; VAW: violence against women.

In a 4-way decomposition analysis, all mediation effects were statistically significant (*P*<.05), whereas none of the interactions reached significance (*P*>.05). Overall, 69.4% of the association between VAW and CDA could be explained by low resilience, with 37.9% of this effect attributable to total mediation. Similarly, 45.2% of the association between VAW and CDA could be explained by low social support, with 25.1% attributable to total mediation (Figure 3 and Table S3 in Multimedia Appendix 1).

Separate mediation and interaction analyses validated similar conclusions. Mediation analysis also showed that low resilience and low social support partially mediated the associations between VAW and mental health outcomes (Table S4 in Multimedia Appendix 1). While the additive and multiplicative interactions of “VAW*resilience” and “VAW*social support” on mental health outcomes were not significant, as the 95% CI of relative excess risk due to interaction crossed 0 and the 95% CI of multiplicative scales crossed 1 (Table S5 in Multimedia Appendix 1).

### Population Attributable Fractions

**Figure 4.**
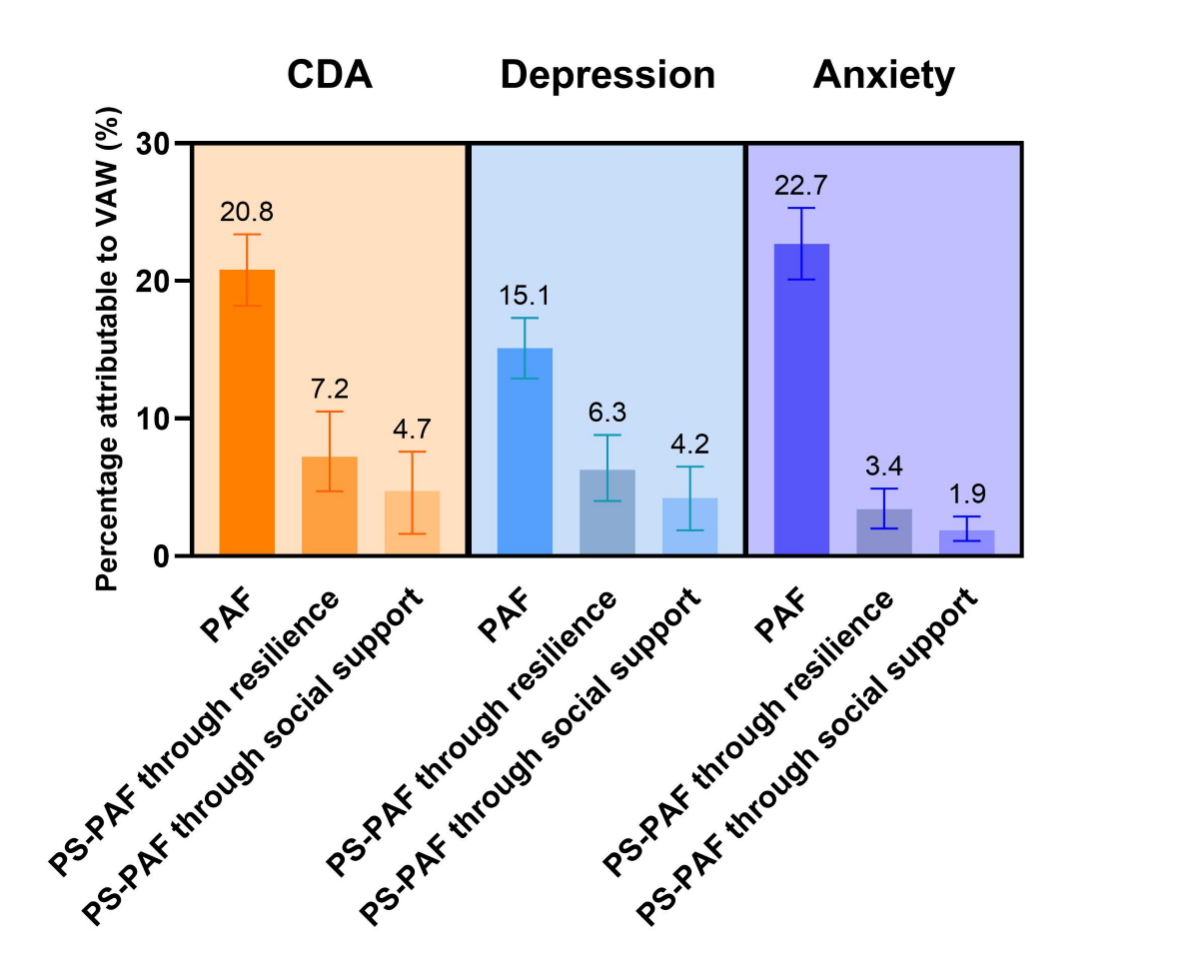
PAF and PS-PAF for violence on mental health outcomes for Chinese rural left-behind women, based on a cross-sectional study conducted in China in 2023. CDA: comorbid symptoms of depression and anxiety; PAF: population attributable fraction; PS-PAF: pathway-specific population attributable fraction; VAW: violence against women.

A total of 20.8% of CDA cases, 15.1% of depressive symptoms cases, and 22.7% of anxiety symptoms cases were attributable to VAW (Figure 4). Among the 3 forms of VAW, the PAF of IPV on depressive symptoms (13.9%) is lower than on anxiety symptoms (23.6%), while the PAF of NPV on depressive symptoms (7.7%) is higher than on anxiety symptoms (5.9%) (Table S6 in Multimedia Appendix 1). The pathway-specific PAFs of CDA, depressive symptoms, and anxiety symptoms attributable to VAW through resilience or social support are presented (Figure 4 and Table S6 in Multimedia Appendix 1). As a mediator, resilience accounted for 7.2%, 6.3%, and 3.4% of CDA, depressive symptoms, and anxiety symptoms with VAW, respectively. The pathway-specific PAFs of social support between VAW and CDA, depressive symptoms, and anxiety symptoms were 4.7%, 4.2%, and 1.9%, respectively.

## Discussion

### Principal Findings

Chinese rural left-behind women’s exposure to lifetime VAW, including IPV (and remote IPV) and NPV, is strongly associated with adverse mental health outcomes, including CDA, depressive, and anxiety symptoms. These associations were partially mediated by low resilience and social support, though interactions were nonsignificant. VAW accounted for a substantial proportion of CDA, depressive, and anxiety symptoms, with differing attributable fractions for IPV and NPV, and resilience and social support partially mediated these attributable proportions.

This study aligns with previous research, reinforcing the association between lifetime VAW and adverse mental health outcomes, including its cumulative effects [44,45]. The left-behind situation among women in rural areas has lasted several decades in China due to disproportionate male labor migration to advanced economic regions. In such a context, women are particularly vulnerable without support from their husbands. Stereotypical gender norms and prevailing beliefs that family matters are private in rural areas constrain women’s autonomy and perpetuate violence [46,47]. Moreover, this vulnerability can be further exacerbated by women’s economic dependence and limited mental health services resources, further deepening their mental health issues [48]. These associations between VAW and mental health issues can also be supported by biological evidence. Repeated violence exposure may lead to dysregulation of the hypothalamic-pituitary-adrenal axis, a critical pathway in the body’s stress response system, contributing to long-term adverse health effects [49]. Moreover, violence exposure can alter neural circuits involved in emotional learning, resulting in impaired memory, heightened threat vigilance, and exaggerated emotional responses to environmental stimuli [50].

The increased risk of adverse mental health outcomes for women exposed to lifetime IPV and NPV is well established and extended by our findings [20,51,52]. Remote IPV has also been found to be an important factor affecting left-behind women’s mental health, calling attention to technology-facilitated remote IPV [16,53]. All IPV types, except economic IPV, were significantly associated with mental health outcomes. The lack of a significant association for economic IPV might be due to the socioeconomic context of rural left-behind women, many of whom are in lower economic strata and depend primarily on their husbands for financial support. Additionally, social and technology-facilitated remote IPV, along with economic and emotional NPV, was associated with higher CDA odds. Our findings expand previous research by comprehensively assessing a broader range of VAW forms, types, and their associations with mental health outcomes.

The associations between VAW and mental health outcomes were partially mediated by low resilience and social support, aligning with previous research. Resilience is thought to buffer the psychological effects of violence, given its ability to access various resources to adapt positively in the face of adversity [54,55]. A study found that empowering women to develop resilience-based coping strategies significantly mediated the impact of IPV on mental health outcomes [56]. Social support is also a vital source of empowerment for women provided by a network of relationships, which helps them cope with stress and maintain well-being. Consistent with our findings, research shows that higher social support buffers the negative impacts of adverse events, helping individuals maintain physical and mental well-being [57,58]. This highlights the importance of strengthening social support networks and fostering resilience as potential interventions to reduce the psychological burden of VAW among rural left-behind women.

Our study estimated that 20.8% of CDA cases, 15.1% of depressive symptoms cases, and 22.7% of anxiety symptoms cases were attributable to VAW. Depressive symptoms were predominantly attributed to IPV, while anxiety symptoms were mainly attributed to NPV. These findings underscore the significant mental health burden associated with VAW, emphasizing the need for clinicians, psychotherapists, and social workers to recognize it as a critical risk factor in rural left-behind women with depression or anxiety. Additionally, pathway-specific PAF revealed that psychological resilience accounted for a greater fraction of mental health outcomes attributable to VAW than social support. This suggests that enhancing resilience may be more effective in reducing the mental health impact of VAW than solely focusing on social support. These findings underscore the importance of incorporating both resilience and social support into public health interventions to address the mental health consequences of VAW.

### Implications for Policy

This study highlights the urgent need for targeted strategies to reduce VAW, foster resilience, and strengthen social support networks through collaborative efforts from communities, health services, and local governments. Such strategies can help to improve mental health outcomes for rural left-behind women in China. For example, implementing educational campaigns to raise prevention awareness of VAW, providing psychological skills training to build resilience, and establishing women’s support groups to strengthen social networks might be practical and effective community-based strategies. Besides, we call for early detection of various forms of VAW and routine mental health screening in both community and health care settings, which can facilitate timely identification and support, such as psychological counseling. Moreover, consistently implementing poverty alleviation and economic empowerment for women living in rural areas in China can further enhance women’s resilience.

### Future Research

Future research should explore prevention and intervention strategies for addressing VAW among left-behind women in rural China. Additionally, intervention studies evaluating the effectiveness of such strategies and policies would provide actionable guidance for improving the mental health of women experiencing violence.

### Limitations

This study also has several limitations. First, the focus on rural left-behind women may limit the generalizability. Second, the use of self-reported data for VAW exposure and covariates introduces potential recall bias, which may underestimate the prevalence of VAW. Third, as a cross-sectional study, this research cannot establish causation, though consistent and cumulative associations suggest potential causal pathways. Finally, the available covariates could not fully capture all relevant factors, such as variables reflecting body fat composition and physical activity, which were not included.

### Conclusions

Our study demonstrates that rural left-behind women who experienced lifetime VAW, including IPV (and remote IPV) and NPV, had significantly higher risks of CDA, depressive and anxiety symptoms, with low resilience and inadequate social support partially mediating these associations. Targeted strategies, including reducing VAW, fostering resilience, and strengthening social support networks, are urgently needed to improve mental health outcomes among rural left-behind women. Future research on the development of VAW prevention and intervention strategies, along with mental health support services, could aid in efforts to mitigate the prevalence of VAW and mental health issues for women in rural areas.

## Supplementary material

10.2196/72064 Multimedia Appendix 1 Supplementary tables and figures.

## Abbreviations

aOR: adjusted odds ratio
CDA: comorbid symptoms of depression and anxiety
IPV: intimate partner violence
NPV: nonpartner violence
OR: odds ratio
PAF: population attributable fraction
RR: risk ratio
VAW: violence against women
