# Supplementary material for: Associations of Violence Against Women With Comorbid Symptoms of Depression and Anxiety Among Left-Behind Women in Rural China: Cross-Sectional Study
Source: JMIR Public Health Surveill. 2025 Aug 7;11:e72064. doi: 10.2196/72064 (PMC12331218; doi:10.2196/72064)
Supplement: Multimedia Appendix 1 [file publichealth-v11-e72064-s001.docx]

**Supplement-Tables and figures**

[Supplements-Methods 2](#_Toc200985035)

[Supplements-Tables 4](#_Toc200985036)

[Table S1. Violence forms and corresponding types and items for Chinese rural left-behind women 4](#_Toc200985037)

[Table S2. Associations between violence and mental health outcomes among Chinese rural left-behind women based on a cross-sectional study conducted in China in 2023 9](#_Toc200985038)

[Table S3. Four-way decomposition of the association of violence and mental health outcomes with low resilience or social support as mediators among Chinese rural left-behind women based on a cross-sectional study conducted in China in 2023 11](#_Toc200985039)

[Table S4. Mediation analysis of adjusted direct and indirect associations between violence and mental health outcomes via resilience, social support among Chinese rural left-behind women based on a cross-sectional study conducted in China in 2023 13](#_Toc200985040)

[Table S5. Additive and multiplicative interactions of violence and resilience, violence and social support with mental health outcomes among Chinese rural left-behind women based on a cross-sectional study conducted in China in 2023 14](#_Toc200985041)

[Table S6. Population attributable fraction and pathway specific population attributable fraction for violence on mental health outcomes for Chinese rural left-behind women based on a cross-sectional study conducted in China in 2023 16](#_Toc200985042)

[Supplements-Figures 17](#_Toc200985043)

[Figure S1. The flow chart of selection for Chinese rural left-behind women from a cross-sectional study conducted in Y city, Henan province, China in 2023 17](#_Toc200985044)

[Figure S2. The dose-risk associations of violence number with mental health outcomes among Chinese rural left-behind women based on a cross-sectional study conducted in China in 2023 18](#_Toc200985045)

Supplements-Methods

**Statistical analysis**

A four-way decomposition analysis was performed to quantify how much of the association between VAW and mental health outcomes could be attributed to mediation (the sum of the mediated interaction and the pure indirect effect) or interaction (the sum of the reference interaction and the mediated interaction) of low resilience and social support[1]. The total effect was decomposed into four distinct quantities. The controlled direct effect is the estimation of the direct effect that is due to neither interaction nor mediation. The reference interaction is the combined effect of VAW and the mediating variables. The mediated interaction is the effect of VAW on mental health outcomes due to mediation and interaction. The pure indirect effect is the effect solely transmitted through the mediator. The total mediation proportion comprises the combined effects of the mediated interaction and the pure indirect effect. Similarly, the total interaction proportion is determined by the sum of the reference interaction and the mediated interaction.

In addition to the decomposition analysis, separate mediation and interaction analyses were were performed to delve deeper into mediated and moderated effect types, thereby further validating the robustness of the findings. Mediation analyses were performed by “Mediation” R package. The joint effects of VAW and resilience, VAW and social support, were evaluated for their interaction on the mental health outcomes through logistic regression. Dummy variables were created for the interaction terms of “VAW*resilience” and “VAW*social support” to examine additive and multiplicative interactions. To estimate the additive scale, the study calculated the relative excess risk due to interaction (RERI): $RERI={OR}_{11}-{OR}_{10}{-OR}_{01}+1$, which can be interpreted as the excess risk resulting from the interaction of two variables compared to the sum of their individual effects[2]. RERI of 0 indicates exact additivity, and thus no interaction on an additive scale; RERI >0 suggests positive interaction or more than additivity; and conversely, RERI<0 indicates negative interaction or less than additivity[2]. To generate the 95% CI of the RERI due to the interaction, we used the delta method of ”InteractionR” R package[3]. The additive interaction metric, attributable proportion (AP) and synergy index (SI), were also derived: AP=$\frac{RERI}{{RR}_{11}}$, SI=$\frac{{RR}_{11}-1}{({RR}_{10}-1)+({RR}_{01}-1)}$ [4]. The multiplicative scale was assessed with the following formula: $multiplicative scale=\frac{{OR}_{11}}{{OR}_{10}\times{OR}_{01}}$[2]. Multiplicative interaction implies that the combined effects of two factors exceed the product of the effects of the two factors considered separately.

**Reference**

1. VanderWeele TJ: A unification of mediation and interaction: a 4-way decomposition. *Epidemiology* 2014, 25(5):749-761.

2. Knol MJ, VanderWeele TJ: Recommendations for presenting analyses of effect modification and interaction. *International journal of epidemiology* 2012, 41(2):514-520.

3. Alli BY: InteractionR: an R package for full reporting of effect modification and interaction. *Software Impacts* 2021, 10:100147.

4. Knol MJ, VanderWeele TJ, Groenwold RH, Klungel OH, Rovers MM, Grobbee DE: Estimating measures of interaction on an additive scale for preventive exposures. *European journal of epidemiology* 2011, 26:433-438.

Supplements-Tables

Table S1. Violence forms and corresponding types and items for Chinese rural left-behind women

| **VAW** | | | |
| --- | --- | --- | --- |
| **Forms** | **Types** | **Items (English)** | **Items (Chinese)** |
| **IPV** |  |  |  |
|  | **Social IPV** | |  |
|  |  | Tried to keep you from seeing your friends | 不让你见您的朋友 |
|  |  | Tried to restrict contact with your family of birth | 限制您与您出生家庭（娘家）的接触 |
|  |  | Insisted on knowing where you are at all times | 坚持要知道您每天的行踪 |
|  |  | Got angry if you speak with another man | 会因为您与其他男性讲话而生气 |
|  |  | Was often suspicious that you are unfaithful | 经常怀疑您不忠诚 |
|  |  | Expected you to ask his permission before seeking health care for yourself | 认为您去医院/诊所之前应当先征得他的同意 |
|  | **Economic IPV** | |  |
|  |  | Prohibited you from getting a job, going to work, trading, earning money or participating in income generation projects | 禁止您找工作、上班、交易、赚钱或参与创收项目? （目的是经济控制，而非关心，注意甄别） |
|  |  | Took your earnings from you against your will | 不经您同意拿走您赚的钱 |
|  |  | Refused to give you money you needed for household expenses even when he has money for other things (such as alcohol and cigarettes) | 即使他有钱买其他东西（如烟和酒），他也拒绝给您家用 |
|  | **Emotional IPV** | |  |
|  |  | Insulted you or made you feel bad about yourself | 侮辱您或让您觉得自己很差劲 |
|  |  | Belittled or humiliated you in front of other people | 在别人面前贬低或羞辱您 |
|  |  | Done things to scare or intimidate you on purpose (e.g. by the way he looked at you, by yelling and smashing things) | 故意做一些事情来吓唬或恐吓您 (例如，他看您的方式，大喊大叫和砸东西) |
|  |  | Verbally threatened to hurt you or someone you care about | 口头威胁要伤害您或您在意的人 |
|  | **Physical IPV** | |  |
|  |  | Slapped you or thrown something at you that could hurt you | 扇您巴掌或朝您扔可能伤害到您的东西 |
|  |  | Pushed you or shoved you or pulled your hair | 推您或扯您头发 |
|  |  | Hit you with his fist or with something else that could hurt you | 用拳头或其他能伤害到您的东西打您 |
|  |  | Kicked you, dragged you or beaten you up | 踢您，拖您或是把您推倒下 |
|  |  | Choked or burnt you on purpose | 故意掐您或烧您 |
|  |  | Threatened with or actually used a knife or other dangerous things against you* | 用刀或其他危险物件威胁您 |
|  | **Sexual IPV** | |  |
|  |  | Forced you to have sexual intercourse when you did not want to, for example by threatening you or holding you down | 在您不想性交的时候强迫您性交，例如威胁您或压制您（如有必要:我们将性交定义为阴道、口腔或肛门插入） |
|  |  | Ever had sexual intercourse you did not want to because you were afraid of what your partner or any other husband or partner might do if you refused | 曾经因为害怕您的丈夫在您拒绝性交后而做什么, 不想进行性行为，却又被迫进行 |
|  |  | Forced you to do anything else sexual that you did not want or that you found degrading or humiliating | 曾经强迫您做任何您不想做的或您觉得有辱人格或令人羞辱的性行为 |
|  | **Technology-facilitated IPV** | |  |
|  |  | Used mobile technology to check where you (are/were) in a way that make you feel controlled | 曾使用追踪软件监控你 |
|  |  | Threatened on digital devices (such as smart phones, laptops, tablets or desktop computers) that he would harm their own bodies if you didn't do as they asked | 曾在数码设备上（如智能手机、笔记本电脑、平板或台式电脑）威胁称如果你不按照他们要求的做就伤害他们自己的身体 |
|  |  | Threatened on digital devices (such as smartphones, laptops, tablets or desktop computers) that he would physically harm your family members or friends | 曾在数码设备上（如智能手机、笔记本电脑、平板或台式电脑）威胁说会对你的家人或朋友进行人身伤害 |
| **Remote-IPV** | |  |  |
|  | **Social remote-IPV(When the partner is out for work)** | |  |
|  |  | Tried to keep you from seeing your friends | 不让你见您的朋友 |
|  |  | Tried to restrict contact with your family of birth | 限制您与您出生家庭（娘家）的接触 |
|  |  | Insisted on knowing where you are at all times | 坚持要知道您每天的行踪 |
|  |  | Got angry if you speak with another man | 会因为您与其他男性讲话而生气 |
|  |  | Was often suspicious that you are unfaithful | 经常怀疑您不忠诚 |
|  |  | Expected you to ask his permission before seeking health care for yourself | 认为您去医院/诊所之前应当先征得他的同意 |
|  | **Economic remote-IPV (When the partner is out for work)** | |  |
|  |  | Prohibited you from getting a job, going to work, trading, earning money or participating in income generation projects | 禁止您找工作、上班、交易、赚钱或参与创收项目? （目的是经济控制，而非关心，注意甄别） |
|  |  | Took your earnings from you against your will | 不经您同意拿走您赚的钱 |
|  |  | Refused to give you money you needed for household expenses even when he has money for other things (such as alcohol and cigarettes) | 即使他有钱买其他东西（如烟和酒），他也拒绝给您家用 |
|  | **Emotional remote-IPV (When the partner is out for work)** | |  |
|  |  | Insulted you or made you feel bad about yourself | 侮辱您或让您觉得自己很差劲 |
|  |  | Belittled or humiliated you in front of other people | 在别人面前贬低或羞辱您 |
|  |  | Done things to scare or intimidate you on purpose (e.g. by the way he looked at you, by yelling and smashing things) | 故意做一些事情来吓唬或恐吓您 (例如，他看您的方式，大喊大叫和砸东西)? |
|  |  | Verbally threatened to hurt you or someone you care about | 口头威胁要伤害您或您在意的人 |
|  | **Technology remote-IPV (When the partner is out for work)** | |  |
|  |  | Used mobile technology to check where you (are/were) in a way that make you feel controlled | 曾使用追踪软件监控你 |
|  |  | Threatened on digital devices (such as smart phones, laptops, tablets or desktop computers) that he would harm their own bodies if you didn't do as they asked | 曾在数码设备上（如智能手机、笔记本电脑、平板或台式电脑）威胁称如果你不按照他们要求的做就伤害他们自己的身体 |
|  |  | Threatened on digital devices (such as smartphones, laptops, tablets or desktop computers) that he would physically harm your family members or friends | 曾在数码设备上（如智能手机、笔记本电脑、平板或台式电脑）威胁说会对你的家人或朋友进行人身伤害 |
| **NPV** |  |  |  |
|  | **Economic NPV** | |  |
|  |  | Prohibited you from getting a job, going to work, trading, earning money or participating in income generation projects | 禁止您找工作、参与家庭以外的创收活动等 |
|  |  | Took your earnings from you against your will | 不经您同意拿走您的钱 |
|  |  | Refused to give you money you needed for household expenses | 限制您的消费行为 |
|  | **Emotional NPV** | |  |
|  |  | Insulted you or made you feel bad about yourself | 侮辱您或让您觉得自己很差劲 |
|  |  | Belittled or humiliated you in front of other people | 在别人面前贬低或羞辱您 |
|  |  | Done things to scare or intimidate you on purpose (e.g. by the way he/she looked at you, by yelling and smashing things) | 故意做一些事情来吓唬或恐吓您 (例如，他看您的方式，大喊大叫和砸东西)? |
|  |  | Verbally threatened to hurt you or someone you care about | 口头威胁要伤害您或您在意的人 |
|  | **Physical NPV** | |  |
|  |  | Slapped you or thrown something at you that could hurt you | 扇您巴掌或朝您扔可能伤害到您的东西 |
|  |  | Pushed you or shoved you or pulled your hair | 推您或扯您头发 |
|  |  | Kicked you, dragged you or beaten you up | 踢您，拖您或是把您推倒下 |
|  |  | Choked or burnt you on purpose | 故意掐您或烧您 |
|  |  | Threatened with or actually used a knife or other dangerous things against you* | 用刀或其他危险物件威胁您 |
|  | **Sexual NPV** | |  |
|  |  | Touched you sexually against your will, or made you do something sexual that you didn’t want to | 违背您的意愿与您发生性接触，包括触摸乳房或私处 |
|  |  | Made you touch their private parts against your will | 强迫您触摸他们的私处 |
|  |  | Forced you into sexual intercourse when you did not want to | 在您不愿意的情况下强迫您发生性关系，如威胁您，按倒您，或者让您处于无法拒绝的境地 |
|  |  | Forced you to have sex when you were too drunk or drugged to refuse | 在您喝醉或意识不清而无法拒绝的情况下强迫您发生性行为 |
|  | **Social NPV** | |  |
|  |  | Tried to keep you from seeing your friends | 不让您见自己的朋友 |
|  |  | Tried to restrict contact with your family of birth | 限制您与您出生家庭（娘家）的接触 |
|  |  | Insisted on knowing where you are at all times | 坚持要知道您每天的行踪 |

Notes: VAW, violence against women; IPV, intimate partner violence; remote-IPV, remote intimate partner violence; NPV, non-partner violence.

*as private gun ownership is illegal in China, we have removed “gun” in the question.

Table S2. Associations between violence and mental health outcomes among Chinese rural left-behind women based on a cross-sectional study conducted in China in 2023

|  |  | **CDA** | |  | **Depressive symptom** | |  | **Anxiety symptom** | |
| --- | --- | --- | --- | --- | --- | --- | --- | --- | --- |
|  |  | **OR (95%CI)** | ***P*-value** |  | **OR (95%CI)** | ***P*-value** |  | **OR (95%CI)** | ***P*-value** |
| **VAW** | |  |  |  |  |  |  |  |  |
| **Any VAW** | |  |  |  |  |  |  |  |  |
|  | No | Ref |  |  | Ref |  |  | Ref |  |
|  | VAW | 1.84 (1.32, 2.54) | <0.001 |  | 1.57 (1.16, 2.10) | 0.003 |  | 1.94 (1.53, 2.46) | <0.001 |
| **VAW number** | | 1.21 (1.13, 1.29) | <0.001 |  | 1.18 (1.11, 1.26) | <0.001 |  | 1.17 (1.10, 1.24) | <0.001 |
| **Class of VAW number** | |  |  |  |  |  |  |  |  |
|  | 0 | Ref |  |  | Ref |  |  | Ref |  |
|  | 1 | 0.87 (0.50, 1.45) | 0.601 |  | 0.87 (0.54, 1.37) | 0.566 |  | 1.53 (1.09, 2.13) | 0.014 |
|  | 2 | 1.68 (0.90, 2.98) | 0.089 |  | 1.55 (0.88, 2.62) | 0.112 |  | 1.62 (1.03, 2.51) | 0.033 |
|  | >=3 | 3.41 (2.25, 5.15) | <0.001 |  | 2.58 (1.74, 3.80) | <0.001 |  | 2.69 (1.91, 3.78) | <0.001 |
| **IPV** | |  |  |  |  |  |  |  |  |
| **Any IPV** | |  |  |  |  |  |  |  |  |
|  | No | Ref |  |  | Ref |  |  | Ref |  |
|  | IPV | 1.84 (1.32, 2.56) | <0.001 |  | 1.57 (1.16, 2.11) | 0.004 |  | 2.09 (1.64, 2.67) | <0.001 |
| **IPV type (Ref: no corresponding type)** | | |  |  |  |  |  |  |  |
|  | Social IPV | 2.66 (1.56, 4.44) | <0.001 |  | 2.45 (1.48, 3.98) | <0.001 |  | 1.83 (1.16, 2.87) | 0.008 |
|  | Economic IPV | 1.35 (0.55, 2.97) | 0.486 |  | 1.40 (0.62, 2.92) | 0.392 |  | 1.34 (0.68, 2.58) | 0.387 |
|  | Emotional IPV | 2.05 (1.35, 3.07) | 0.001 |  | 1.69 (1.14, 2.48) | 0.007 |  | 2.45 (1.78, 3.39) | <0.001 |
|  | Physical IPV | 2.35 (1.64, 3.34) | <0.001 |  | 1.88 (1.35, 2.60) | <0.001 |  | 1.91 (1.45, 2.50) | <0.001 |
|  | Sexual IPV | 2.79 (1.20, 6.10) | 0.013 |  | 2.43 (1.08, 5.19) | 0.026 |  | 1.85 (0.89, 3.84) | 0.097 |
|  | Technology IPV | 5.95 (1.07, 30.12) | 0.03 |  | 3.97 (0.72, 19.79) | 0.09 |  | 14.04 (2.26, 270.94) | 0.016 |
| **Remote-IPV** | |  |  |  |  |  |  |  |  |
| **Any remote-IPV** | |  |  |  |  |  |  |  |  |
|  | No | Ref |  |  | Ref |  |  | Ref |  |
|  | Remote IPV | 2.79 (1.60, 4.74) | <0.001 |  | 2.44 (1.44, 4.05) | 0.001 |  | 2.46 (1.54, 3.94) | <0.001 |
| **Remote-IPV type (Ref: no corresponding type)** | | |  |  |  |  |  |  |  |
|  | Social remote-IPV | 3.39 (1.71, 6.48) | <0.001 |  | 3.31 (1.74, 6.14) | <0.001 |  | 2.31 (1.28, 4.17) | 0.005 |
|  | Economic remote-IPV | 1.30 (0.36, 3.74) | 0.65 |  | 1.66 (0.57, 4.28) | 0.318 |  | 1.12 (0.44, 2.66) | 0.806 |
|  | Emotional remote-IPV | 1.87 (0.65, 4.72) | 0.208 |  | 2.12 (0.83, 5.00) | 0.098 |  | 2.09 (0.92, 4.72) | 0.074 |
|  | Technology remote-IPV | 5.95 (1.07, 30.12) | 0.03 |  | 3.97 (0.72, 19.79) | 0.09 |  | 14.04 (2.26, 270.94) | 0.016 |
| **NPV** | |  |  |  |  |  |  |  |  |
| **Any NPV** | |  |  |  |  |  |  |  |  |
|  | No | Ref |  |  | Ref |  |  | Ref |  |
|  | NPV | 2.63 (1.58, 4.26) | <0.001 |  | 2.19 (1.36, 3.48) | 0.001 |  | 1.89 (1.24, 2.87) | 0.003 |
| **NPV type (Ref: no corresponding type)** | | |  |  |  |  |  |  |  |
|  | Economic NPV | 6.10 (1.19, 26.48) | 0.017 |  | 7.02 (1.58, 31.3) | 0.008 |  | 2.73 (0.63, 11.85) | 0.163 |
|  | Emotional NPV | 3.15 (1.86, 5.24) | <0.001 |  | 2.37 (1.42, 3.88) | 0.001 |  | 2.08 (1.32, 3.25) | 0.001 |
|  | Physical NPV | 2.44 (0.84, 6.27) | 0.077 |  | 1.80 (0.62, 4.54) | 0.239 |  | 2.76 (1.19, 6.54) | 0.018 |
|  | Sexual NPV | NA | 0.984 |  | NA | 0.964 |  | NA | 0.972 |
|  | Social NPV | NA | 0.976 |  | NA | 0.976 |  | NA | 0.973 |

Notes: CDA, comorbid symptoms of depression and anxiety; OR, odds ratio; CI, confidence interval; VAW, violence against women; IPV, intimate partner violence; remote-IPV, remote intimate partner violence; NPV, non-partner violence.

All models were adjusted for age, education, occupation, household income, unhealthy lifestyle (smoking, drinking, poor sleep quality), BMI, chronic disease history, length of left-behind. OR and 95% CI in red indicate a significant positive association, whereas OR and 95% CI in blue indicate a significant negative association.

Table S3. Four-way decomposition of the association of violence and mental health outcomes with low resilience or social support as mediators among Chinese rural left-behind women based on a cross-sectional study conducted in China in 2023

|  | | **CDA** | | |  | **Depressive symptom** | | |  | **Anxiety symptom** | | |
| --- | --- | --- | --- | --- | --- | --- | --- | --- | --- | --- | --- | --- |
|  |  | **Estimate (95%CI)** | ***P-*value** | **Proportion (95%CI)** |  | **Estimate (95%CI)** | ***P-*value** | **Proportion (95%CI)** |  | **Estimate (95%CI)** | ***P-*value** | **Proportion (95%CI)** |
| **Exposure: VAW** | |  |  |  |  |  |  |  |  |  |  |  |
| **Mediator/moderator: low resilience** | |  |  |  |  |  |  |  |  |  |  |  |
|  | Total effect | **0.83 (0.57, 1.28)** | **0.011** | **100** |  | **0.56 (0.02, 0.86)** | **0.028** | **100** |  | **0.94 (0.72, 1.07)** | **<0.001** | **100** |
|  | Controlled direct effect | 0.25 (0.04, 0.34) | 0.160 | 30.6 (-54.9, 43.3) |  | 0.10 (-0.30, 0.13) | 0.475 | 17.3 (-25.8, 23.9) |  | **0.54 (0.37, 0.57)** | **0.007** | **57.5 (43.6, 62.0)** |
|  | Reference interaction | 0.26 (0.06, 0.75) | 0.263 | 31.5 (14.6, 123.3) |  | 0.19 (-0.02, 0.44) | 0.310 | 34.1 (25.3, 87.9) |  | 0.20 (0.08, 0.31) | 0.266 | 21.6 (9.2, 32.1) |
|  | Mediated interaction | 0.08 (0.04, 0.21) | 0.278 | 9.7 (6.3, 33.7) |  | 0.06 (0.00, 0.13) | 0.323 | 10.5 (06.7, 25.7) |  | 0.06 (0.04, 0.10) | 0.279 | 6.6 (5.2, 10.6) |
|  | Pure indirect effect | **0.23 (0.23, 0.33)** | **<0.001** | **28.2 (-2.0, 41.5)** |  | **0.21 (0.22, 0.29)** | **<0.001** | **38.0 (12.2, 63.7)** |  | **0.13 (0.12, 0.20)** | **<0.001** | **14.3 (13.7, 21.5)** |
| **Mediator/moderator: low social support** | |  |  |  |  |  |  |  |  |  |  |  |
|  | Total effect | **0.78 (0.50, 1.19)** | **0.010** | **100** |  | **0.52 (0.07, 0.80)** | **0.028** | **100** |  | **0.92 (0.74, 1.00)** | **<0.001** | **100** |
|  | Controlled direct effect | 0.43 (0.16, 0.65) | 0.111 | 54.8 (38.0, 53.0) |  | 0.33 (-0.40, 0.37) | 0.143 | 62.7 (-7.5, 73.1) |  | **0.72 (0.09, 0.94)** | **0.004** | **78.0 (24.9, 111.4)** |
|  | Reference interaction | 0.16 (0.09, 0.31) | 0.464 | 20.1 (19.3, 39.6) |  | 0.04 (-0.06, 0.26) | 0.800 | 8.4 (-1.7, 79.5) |  | 0.09 (-0.22, 0.50) | 0.609 | 9.5 (-16.8, 50.8) |
|  | Mediated interaction | 0.05 (0.03, 0.10) | 0.470 | 6.5 (5.9, 12.8) |  | 0.01 (-0.02, 0.10) | 0.801 | 2.7 (-0.8, 20.6) |  | 0.03 (-0.08, 0.19) | 0.611 | 3.1 (-6.2, 19.1) |
|  | Pure indirect effect | **0.15 (0.07, 0.16)** | **<0.001** | **18.6 (0.4, 25.1)** |  | **0.14 (0.07, 0.16)** | **<0.001** | **26.2 (7.3, 37.4)** |  | **0.09 (0.03, 0.10)** | **0.001** | **9.5 (4.1, 11.6)** |

Notes: CDA, co-occurrence of depressive and anxiety symptoms; VAW, violence against women. Controlled direct effect indicates association due neither to mediation or interaction, reference interaction indicates association due to interaction only, mediated interaction indicates association due to mediation and interaction, pure indirect effect indicates association due to mediation only.

All models were adjusted for age, education, occupation, household income, unhealthy lifestyle (smoking, drinking, poor sleep quality), BMI, chronic disease history, length of left-behind.

Table S4. Mediation analysis of adjusted direct and indirect associations between violence and mental health outcomes via resilience, social support among Chinese rural left-behind women based on a cross-sectional study conducted in China in 2023

|  |  | **CDA** | |  | **Depressive symptom** | |  | **Anxiety symptom** | |
| --- | --- | --- | --- | --- | --- | --- | --- | --- | --- |
|  |  | **OR (95%CI) or Estimate (95%CI)** | ***P*-value** |  | **OR (95%CI) or Estimate (95%CI)** | ***P*-value** |  | **OR (95%CI) or Estimate (95%CI)** | ***P*-value** |
| **VAW->Resilience->Outcome** | |  |  |  |  |  |  |  |  |
|  | VAW->Resilience | 0.13 (0.06-0.31) | <0.001 |  | 0.13 (0.06-0.31) | <0.001 |  | 0.13 (0.06-0.31) | <0.001 |
|  | Resilience->Outcome | 0.88 (0.86-0.90) | <0.001 |  | 0.88 (0.86-0.90) | <0.001 |  | 0.93 (0.91-0.94) | <0.001 |
|  | VAW->Outcome | 1.58 (1.11-2.24) | 0.01 |  | 1.32 (0.96-1.82) | 0.089 |  | 1.75 (1.36-2.24) | <0.001 |
|  | ACME | 0.022 (0.012-0.035) | <0.001 |  | 0.028 (0.015-0.039) | <0.001 |  | 0.028 (0.014-0.04) | <0.001 |
|  | ADE | 0.042 (0.014-0.073) | <0.001 |  | 0.03 (-0.005-0.067) | 0.1 |  | 0.107 (0.056-0.151) | <0.001 |
|  | TE | 0.07 (0.037-0.099) | <0.001 |  | 0.062 (0.019-0.114) | <0.001 |  | 0.14 (0.085-0.188) | <0.001 |
|  | Mediated proportion (%) | 31.59 (16.6-61.8) | <0.001 |  | 44.77 (25.77-136.48) | <0.001 |  | 19.88 (10.73-34.24) | <0.001 |
| **VAW->Social support->Outcome** | |  |  |  |  |  |  |  |  |
|  | VAW->Social support | 0.17 (0.09-0.33) | <0.001 |  | 0.17 (0.09-0.33) | <0.001 |  | 0.17 (0.09-0.33) | <0.001 |
|  | Social support->Outcome | 0.92 (0.89-0.94) | <0.001 |  | 0.92 (0.89-0.94) | <0.001 |  | 0.95 (0.94-0.97) | <0.001 |
|  | VAW->Outcome | 1.54 (1.10-2.16) | 0.011 |  | 1.32 (0.97-1.79) | 0.078 |  | 1.79 (1.41-2.29) | <0.001 |
|  | ACME | 0.014 (0.008-0.022) | <0.001 |  | 0.018 (0.011-0.027) | <0.001 |  | 0.015 (0.008-0.025) | <0.001 |
|  | ADE | 0.044 (0.007-0.082) | <0.001 |  | 0.034 (-0.01-0.068) | 0.08 |  | 0.118 (0.068-0.166) | <0.001 |
|  | TE | 0.062 (0.023-0.10) | <0.001 |  | 0.055 (0.009-0.096) | <0.001 |  | 0.137 (0.086-0.183) | <0.001 |
|  | Mediated proportion (%) | 22.5 (10.23-66.9) | <0.001 |  | 33.21 (16.43-226.65) | <0.001 |  | 11.26 (5.99-21.51) | <0.001 |

Notes: CDA, comorbid symptoms of depression and anxiety; VAW, violence against women; OR, odds ratio; CI, confidence interval; SE, standard error; ACME, average causal mediation effects; ADE, average direct effects; TE, total effect; MP, mediated proportion.

All models were adjusted for age, education, occupation, household income, unhealthy lifestyle (smoking, drinking, poor sleep quality), BMI, chronic disease history, length of left-behind.

Table S5. Additive and multiplicative interactions of violence and resilience, violence and social support with mental health outcomes among Chinese rural left-behind women based on a cross-sectional study conducted in China in 2023

|  |  | **CDA** | |  | **Depressive symptom** | |  | **Anxiety symptom** | |
| --- | --- | --- | --- | --- | --- | --- | --- | --- | --- |
|  |  | **OR (95% CI)** | **Estimate (95%CI)** |  | **OR (95% CI)** | **Estimate (95%CI)** |  | **OR (95% CI)** | **Estimate (95%CI)** |
| **VAW*Resilience** | |  |  |  |  |  |  |  |  |
|  | VAW (yes) *Resilience (low) | 12.24 (6.75, 22.19) |  |  | 8.39 (5.20, 13.54) |  |  | 4.61 (3.30, 6.45) |  |
|  | VAW (yes) *Resilience (high) | 2.10 (0.92, 4.79) |  |  | 1.35 (0.66, 2.74) |  |  | 1.97 (1.34, 2.90) |  |
|  | VAW (no) *Resilience (low) | 8.38 (4.74, 14.83) |  |  | 6.41 (4.09, 10.04) |  |  | 2.78 (2.07, 3.75) |  |
|  | VAW (no) *Resilience (high) | 1 (Reference) |  |  | 1 (Reference) |  |  | 1 (Reference) |  |
|  | **Multiplicative interaction** |  | 0.70 (0.28, 1.72) |  |  | 0.97 (0.44, 2.13) |  |  | 0.84 (0.51, 1.38) |
|  | **RERI** |  | 2.76 (-2.31, 9.65) |  |  | 1.63 (-1.41, 5.28) |  |  | 0.86 (-0.57, 2.41) |
|  | **AP** |  | 0.23 (-0.16, 0.46) |  |  | 0.19 (-0.17, 0.43) |  |  | 0.19 (-0.15, 0.41) |
|  | **SI** |  | 1.33 (0.87, 2.02) |  |  | 1.28 (0.84, 1.95) |  |  | 1.31 (0.84, 2.04) |
| **VAW*Social support** | |  |  |  |  |  |  |  |  |
|  | VAW (yes) *Social support (low) | 4.45 (2.82, 7.03) |  |  | 3.52 (2.34, 5.29) |  |  | 3.17 (2.29, 4.38) |  |
|  | VAW (yes) *Social support (high) | 1.82 (1.01, 3.27) |  |  | 1.60 (0.96, 2.67) |  |  | 2.00 (1.39, 2.88) |  |
|  | VAW (no) *Social support (low) | 2.95 (1.91, 4.57) |  |  | 2.76 (1.89, 4.03) |  |  | 1.90 (1.42, 2.54) |  |
|  | VAW (no) *Social support (high) | 1 (Reference) |  |  | 1 (Reference) |  |  | 1 (Reference) |  |
|  | **Multiplicative interaction** |  | 0.83 (0.41, 1.68) |  |  | 0.80 (0.42, 1.50) |  |  | 0.83 (0.51, 1.36) |
|  | **RERI** |  | 0.68 (-1.39, 2.70) |  |  | 0.16 (-1.47, 1.63) |  |  | 0.27 (-0.89, 1.38) |
|  | **AP** |  | 0.15 (-0.34, 0.44) |  |  | 0.05 (-0.46, 0.35) |  |  | 0.08 (-0.33, 0.35) |
|  | **SI** |  | 1.24 (0.69, 2.24) |  |  | 1.07 (0.60, 1.91) |  |  | 1.14 (0.67, 1.95) |

Notes: CDA, comorbid symptoms of depression and anxiety; VAW, violence against women; OR, odds ratio; CI, confidence interval; RERI, relative excess risk due to interaction; AP, attributable proportion due to additive interaction; SI, synergy index.

All models were adjusted for age, education, occupation, household income, unhealthy lifestyle (smoking, drinking, poor sleep quality), BMI, chronic disease history, length of left-behind.

Table S6. Population attributable fraction and pathway specific population attributable fraction for violence on mental health outcomes for Chinese rural left-behind women based on a cross-sectional study conducted in China in 2023

|  | **CDA** | | |  | **Depressive symptom** | | |  | **Anxiety symptom** | | |
| --- | --- | --- | --- | --- | --- | --- | --- | --- | --- | --- | --- |
|  | **PAF% (95% CI)** | **PS-PAF% (95% CI)  through resilience** | **PS-PAF% (95% CI)  through social support** |  | **PAF% (95% CI)** | **PS-PAF% (95% CI)  through resilience** | **PS-PAF% (95% CI)  through social support** |  | **PAF% (95% CI)** | **PS-PAF% (95% CI)  through resilience** | **PS-PAF% (95% CI)  through social support** |
| VAW | 20.8 (18.2, 23.4) | 7.2 (4.7, 10.5) | 4.7 (1.6, 7.6) |  | 15.1 (12.9, 17.3) | 6.3 (4, 8.8) | 4.2 (1.9, 6.5) |  | 22.7 (20.1, 25.3) | 3.4 (2, 4.9) | 1.9 (1.1, 2.9) |
| IPV | 19.2 (16.7, 21.7) | 6.5 (3.7, 9.7) | 3.8 (1.6, 5.7) |  | 13.9 (11.7, 16.1) | 5.6 (2.9, 8) | 3.4 (1.8, 5) |  | 23.6 (20.9, 26.3) | 3.1 (1.5, 4.7) | 1.6 (0.7, 2.5) |
| Remote-IPV | 8.8 (7.0, 10.6) | 1.3 (-0.3, 3) | 1.1 (0.1, 2.1) |  | 7.2 (5.6, 8.8) | 1.1 (0.2, 2.1) | 1 (0.2, 2) |  | 7.3 (5.7, 8.9) | 0.5 (0, 0.9) | 0.4 (0.1, 0.8) |
| NPV | 10.2 (8.3, 12.1) | 2.4 (0.8, 4.1) | 2.5 (0.8, 4.1) |  | 7.7 (6.1, 9.3) | 2 (1.2, 3) | 2.1 (1.1, 3.1) |  | 5.9 (4.4, 7.4) | 0.9 (0.3, 1.6) | 0.9 (0.3, 1.4) |

Notes: CDA, comorbid symptoms of depression and anxiety; VAW, violence against women; PAF, population attributable fraction; PS-PAF, pathway specific population attributable fraction.

Supplements-Figures


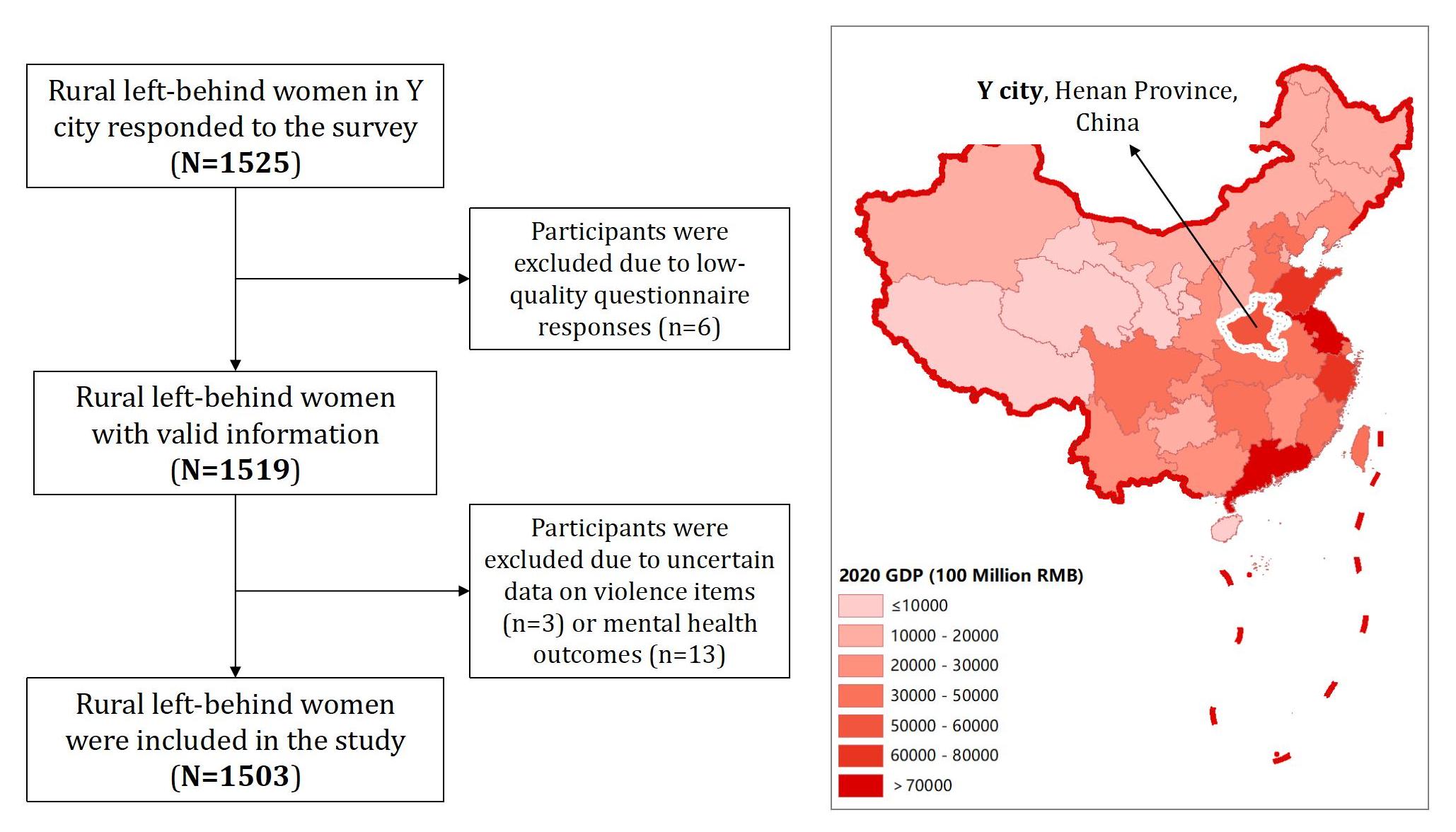


Figure S1. The flow chart of selection for Chinese rural left-behind women from a cross-sectional study conducted in Y city, Henan province, China in 2023

Notes: GDP, gross domestic product


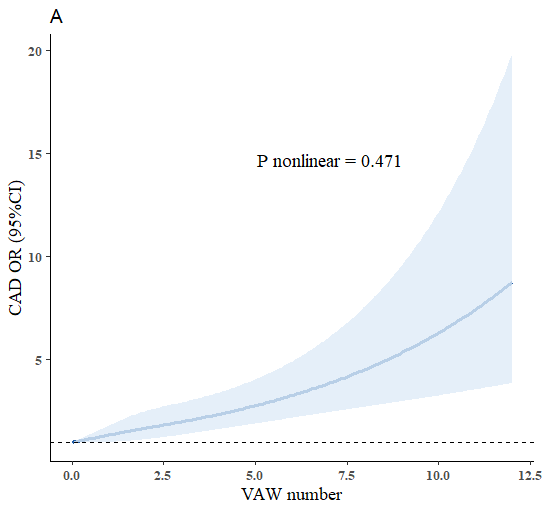

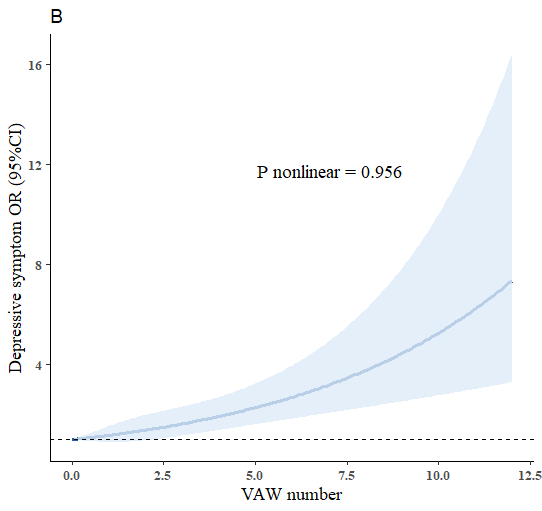

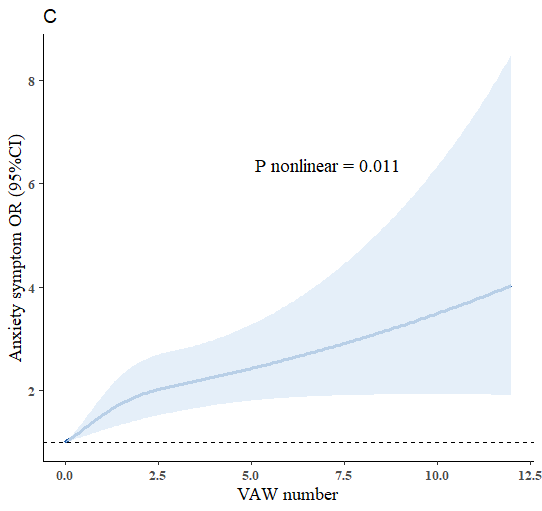


Figure S2. The dose-risk associations of violence number with mental health outcomes among Chinese rural left-behind women based on a cross-sectional study conducted in China in 2023

Notes: CDA, comorbid symptoms of depression and anxiety; VAW, violence against women; OR, odds ratio; CI, confidence interval.

Model was adjusted for age, education, occupation, household income, unhealthy lifestyle (smoking, drinking, poor sleep quality), BMI, chronic disease history, length of left-behind.
